# Relationship between empathy and motivation in undergraduate medical students

**DOI:** 10.3205/zma001336

**Published:** 2020-06-15

**Authors:** Ardi Findyartini, Estivana Felaza, Daniar Setyorini, Rita Mustika

**Affiliations:** 1 University Indonesia, Faculty of Medicine, Department of Medical Education, Jakarta, Indonesia; 2 University Indonesia, Faculty of Medicine, Medical Education Center, Indonesian Medical Education and Research Institute (IMERI), Jakarta, Indonesia

**Keywords:** undergraduate medical students, empathy, motivation

## Abstract

**Background: **Undergraduate medical education is important for encouraging empathy which is a critical component of patient-physician communication. Studies show a decline in empathy once medical students enter their clinical years. Since empathy is also a “motivated phenomenon”, the current study aims to explore the relationship between empathy and students’ motivation types.

**Methods: **This cross-sectional study used a total sampling approach to recruit medical students in years 1-5. The Jefferson Scale of Physician Empathy (JSPE) was used to measure empathy in medical students and the Academic Motivation Scale (AMS) was utilised to assess student motivation. Following descriptive analyses, the differences in empathy scores based on motivation type was assessed using Kruskal-Wallis test and post-hoc Mann-Whitney test. Furthermore, the Spearman’s rank correlation analysis was completed to assess the relationship between students’ empathy and motivation type. The analyses were completed for each of year 1-5.

**Results: **A total of 827 completed questionnaires (71.3% response rate) were analysed, showing strong internal consistency. Most students displayed high intrinsic and high controlled motivation. Motivation type was found to be consistently associated with empathy.

**Conclusions: **The present study highlights the association of motivation with empathy in undergraduate medical students with an increasingly low empathy score the more the motivation profile is towards being Low Intrinsic and Low Controlled.

## Zusammenfassung

**Hintergrund:** Die medizinische Ausbildung im Bachelorstudiengang ist wichtig, um die Empathie (das Einfühlungsvermögen) zu fördern. Sie ist eine kritische Komponente in der Kommunikation zwischen Patienten und Arzt. Studien zeigen jedoch einen Rückgang der Empathie, sobald Medizinstudenten in ihre klinischen Jahre eintreten. Weil Empathie auch ein „motiviertes Phänomen“ ist, zielt die aktuelle Studie darauf ab, die Beziehung zwischen Empathie und Motivationsart der Studenten zu untersuchen.

**Methoden: **Diese Querschnittsstudie verwendete einen Gesamtstichprobenansatz, um Medizinstudenten zwischen dem 1. und 5. Studienjahr anzuwerben. Die Jefferson Scale of Physician Empathy (JSPE) wurde angewandt, um die Empathie der Medizinstudenten zu vermessen und die Academic Motivation Scale (AMS), um deren Motivation zu bewerten. Nach den deskriptiven Analysen wurden die Unterschiede in den Empathiewerten (Scores) mithilfe des Kruskal-Wallis-Tests und des Post-hoc-Mann-Whitney-Tests basierend auf der Motivationsart bewertet. Darüber hinaus wurde eine Rangkorrelationsanalyse nach Spearman durchgeführt, um die Beziehung zwischen Empathie und Motivationsarten der Studierenden auszuwerten. Die Analysen wurden für jedes Studienjahr durchgeführt.

**Ergebnisse: **Insgesamt wurden 827 ausgefüllte Fragebögen (71,3% Resonanzquote) analysiert. Diese zeigten eine starke interne Konsistenz. Die Mehrheit der Studierenden zeigten Hoch-Intrinsisch und Hoch-Kontrollierte Motivationen. Es wurde gezeigt, dass die Art der Motivation konsistent mit Empathie verbunden ist.

**Schlussfolgerungen: **Die vorliegende Studie beleuchtet insbesondere die Verbindung zwischen der Motivation und Empathie bei den Medizinstudenten. Je weiter sich das Motivationsprofil in Richtung der Gering-Intrinsischen und Wenig-Kontrollierten Motivation bewegte, desto geringer waren die Empathiewerte.

## Background

Physician empathy has been regarded as a critical component of patient–physician communication, as it leads to greater satisfaction among patients [1], [2] increased participation and education among patients [1], [2], [3], reduced emotional distress and increased quality of life among patients [4], and increased diagnostic accuracy among physicians [5], [6], [7]. A widely accepted definition for empathy, as suggested by Mercer and Reynolds highlights its cognitive, affective and behavioural/action aspects [3]. According to these authors, empathising means to understand the patient’s situation, perspective and feelings; to communicate and check the accuracy of this understanding; and to act based on this understanding in order to help the patient [3]. A systematic review conducted by Sulzer et al. found that of all three components of empathy (i.e. cognitive, affective and behavioural), the cognitive process is the most prevalent concept used, whereas the affective aspect is the least [8]. Several studies used a definition for empathy proposed by Hojat and LaNoue ([9], p. 74), which states that empathy is “a predominantly cognitive attribute that involves an understanding of patients’ experiences, concerns and perspectives combined with a capacity to communicate this understanding”. 

Medical schools in many countries have attempted to develop empathy in their medical students and residents. A systematic review by Neumann et al. [10], focusing on studies with longitudinal data, revealed that empathy declines during medical studies and residencies, particularly during the clinical phase of training, which is thought to be caused by distress in students due to hidden curricula. Various factors are considered to be associated with empathy development, such as burnout [11], the climate of professionalism [12], and motivation [13], [14].

Empathy may vary depending on individual differences [15] and subjective judgments [16], and it can be both automatic and situational [14]. Empathy is “a motivated phenomenon” in which people may choose to experience or avoid the process of understanding other people’s emotions [14]. Some phenomena, such as suffering, material costs and interference from competition, may drive people to avoid empathy, whereas positive affect, affiliation and social desirability may encourage them to become more empathetic [14]. A study by Duan argued that motivation increases intellectual empathy (the cognitive component) when the target person is sad, and empathic emotion (the affective component) when the target person is happy [13].

Learning in medical schools requires adequate motivation, including learning to be empathetic with patients and their families. Based on the self-determination theory [17], motivation can be defined as a continuum between amotivation, in which there is a feeling of incompetency and an inability to obtain a desired outcome, extrinsic motivation, in which the urge to do or complete something is determined by the environment or external factors, and intrinsic motivation, in which the drive to pursue an activity is aimed at personal satisfaction [18]. Four motivational profiles have been introduced by Kusurkar et al. [19], namely High Intrinsic High Controlled (HIHC) type which reflects high intrinsic motivation/interest and controlled/status motivation and High Intrinsic Low Controlled (HILC) type which reflects high intrinsic motivation/interest and low controlled/status motivation. In addition, Low Intrinsic High Controlled (LIHC) type refers to low intrinsic motivation/interest and high controlled/status motivation, whereas both intrinsic motivation/interest and controlled/status motivation are low in the Low Intrinsic Low Controlled (LILC) motivational type. 

To the authors’ best knowledge, there are limited studies exploring the associations between empathy and motivational profiles among medical students across the undergraduate years of study. The present study aimed to explore the relationship between empathy and students’ motivation types. In addition, given the results of previous studies on empathy and motivation [13], [14], the present authors hypothesise that students with high intrinsic and/or high controlled motivation will achieve better empathy scores compared to students with other motivational profiles. The findings of this study are expected to inform empathy development and suggest the ideal learning environment for medical students. Undergraduate medical education represents a critical time for empathy development, considering that this stage has a strong influence on medical students’ professional development, in which they may identify with their future professional roles but have not yet fully integrated them into their practices [20].

## Methods

### Context 

This study was conducted in the undergraduate medical programme at the Faculty of Medicine, Universitas Indonesia (FMUI). The programme is structured with a 5.5-year curriculum, consisting of an academic/preclinical stage (3.5 years) and a clinical stage (2 years). The competency-based curriculum implements an integrated approach, involving the biomedical, clinical and social sciences, as well as humanism. The programme recognises the importance of developing knowledge of the humanism, professionalism and cultural competence, including empathy, by offering structured, longitudinal courses during the preclinical stage. Students are encouraged to understand the basic concepts of empathy, observe real clinical practices and reflect on their empathy development through a series of discussions, reflections, field activities and role-model shadowing. Such structures are implemented in a more integrative way during the clinical stage. The programme also emphasises faculty development to support the roles of medical teachers as resources, facilitators and role models for empathy and professional development.

#### Study design and instruments

This cross-sectional study used total sampling and was completed in May 2018. The Academic Motivation Scale (AMS) [21] and the Jefferson Scale of Physician Empathy (JSPE; student version) [22] were used to measure the students’ motivation and empathy, respectively. All instruments were administered in the Indonesian language. Both instruments have been validated for use Indonesian in the previous studies [23], [24]. 

The AMS questionnaire consists of 28 questions wich were rated on a 7-point Likert scale, ranging from “not related at all” to “very related”. The original version of the questionnaire is comprised of seven subscales: three for intrinsic motivation (i.e. to know, towards accomplishment, to experience stimulation), three for extrinsic motivation (i.e. identified, introjected, external regulation), and amotivation. In the present study, Kusurkar et al.’s framework [19], which reorganised the questionnaire into four types of motivation (i.e. HIHC, HILC, LIHC and LILC), was used. All scores for intrinsic and controlled motivation types were then categorised into high (average of each item >3.5) and low groups (average of each item ≤3.5). The four types were coded as ordinal data from 1-4, reflecting HIHC, HILC, LIHC and LILC, respectively. 

Finally, to measure empathy, this study administered the JSPE for medical students [22]. The instrument focuses on the cognitive aspect of empathy, showing good internal consistency (0.7–0.9) in previous administrations in other languages [22], [25], [26].

#### Sample

A total of 1,160 students were invited to participate in this study. The questionnaires were administered to students in years 1-5 using a total sampling approach, allowing for voluntary participation. By the time the study was conducted, the students were in the second semester of their respective years. 

#### Data collection

Both questionnaires were administered to the entire sample, except for students in their final year, as they had already completed their studies due to the structure of the 5.5-year curriculum. To increase the response rate and validity, the students filled in a hardcopy of the questionnaires following the completion of a Progress Test tailored to each academic year. The students were assured that participation in the study was voluntary and there would be no consequences towards their academic assessment based on their participation. The research team provided a small incentive (i.e. reusable water bottles) to those who completed the questionnaires. 

#### Data analysis

The data was filtered so that only the completed questionnaires were analysed. The analysis was completed using SPSS 22.0. The scores and groups for the AMS and the JSPE were obtained according to the manuals relevant to each measure. The internal consistency of each questionnaire was assessed using Cronbach’s alpha. Descriptive analyses were completed to describe the demographic characteristics and relevant scores of each respondent according to their academic year. All data was analysed for normality, homogeneity and bivariate relationships. Given the abnormal data distribution, the differences in empathy scores based on motivation type in each year was assessed using Kruskal–Wallis test and posthoc Mann-Whitney test. Furthermore, the Spearman’s rank correlation analysis was completed to assess the relationship between students’ empathy and motivation type. All analyses were completed for each year, considering the differences in learning experience and curriculum between years. 

The study was approved by the Research Ethics Committee at the FMUI (No 451/UN2.F1/ETIK/IV/2018). The participants gave written consent and acknowledged that any reports from the present study would be fully anonymised. 

## Results

A total of 1,061 medical students in years 1–5 participated in this study. Following data cleansing, 827 questionnaires (71.3% response rate) were analysed. The internal consistency as measured by Cronbach’s alpha of all questionnaires was very good (AMS: 0.794; JSPE: 0.794). 

The characteristics of the respondents are described in table 1 (Tab. 1). The proportion of female students was higher than the proportion of male students across all years. Most students who had high intrinsic and high controlled motivation, showing higher score of empathy up to the third preclinical year (year 3), a slightly lower score in the first clinical year (year 4) and a further lower score in the second clinical year (year 5) (Kruskal-Wallis: χ^2^ 18,262, df 4, p=0.001). Post-hoc Mann-Whitney tests revealed that differences of empathy scores were significant between the following groups:

1. Preclinical Year 1 and 2 (Mann-Whitney U 10852,000, p=0.037)
2. Preclinical Year 1 and 3 (Mann-Whitney U 10555,000, p=0.000)
3. Preclinical Year 1 and clinical Year 1/Year 4 (Mann-Whitney U 11284,500, p=0.002)
4. Preclinical Year 2 and clinical Year 2/Year 5 (Mann-Whitney U 8461,000, p=0.015)
5. Preclinical Year 3 and clinical Year 2/Year 5 (Mann-Whitney U 8183,000, p=0.000)
6. Clinical year 1 and clinical Year 2/Year 5 (Mann-Whitney U 8855,000, p=0.001)

Across year levels, the minimum mean of empathy score was 114.38±12.76 in first-year preclinical students, and the maximum was 119.58±9.52 in third-year preclinical students. 

Table 2 (Tab. 2) describes the empathy score according to motivation type. The Kruskal-Wallis test results suggest that there are statistically significant differences in empathy scores based on motivation types in first- and third-year preclinical students (years 1 and 3) and second-year clinical students (year 5). The Mann-Whitney test revealed statistically significant differences in empathy scores in first-year preclinical students (year 1) between the HIHC and HILC types (Mann-Whitney U 646,500; p=0.022), in third-year preclinical students (year 3) between the HIHC/HILC and LIHC types (Mann-Whitney U 421,000; p=0.012 and Mann-Whitney U 20,000; p=0.011, respectively), and between the LIHC and LILC types (Mann-Whitney U 3,500; p=0.008); and in second-year clinical students (year 5) between HIHC and LILC types (Mann-Whitney U 216,000; p=0.004). 

Spearman’s rank correlation analysis was completed for each year to explore the possible associations between empathy and motivation type (see table 3 (Tab. 3)). Table 3 (Tab. 3) shows that motivation type seems to be negatively low correlated with empathy, but only among first- and second-year preclinical students and second-year clinical students (year 5). For this analysis, the motivation profiles were arranged accordingly from HIHC, HILC to LIHC and LILC motivational types and they were coded from 1-4 respectively. Therefore, the negative correlation means that the closer the motivation profile is towards LILC (coded as 4), the lower the empathy score. 

## Discussion

The present study was aimed at exploring the relationship between empathy and motivation type among medical students across years 1-5 of their undergraduate studies. In addition to the study by Duan which investigated the relationship between empathy and motivation in an experimental setting [13], to the authors’ best knowledge, this is the first study exploring the association between medical students’ academic motivation and empathy. As suggested by Zaki [14], empathy is a motivated phenomenon in which social desirability and intrinsic motivation interact. 

Overall, the mean empathy scores of the medical students in this study (ranging from 114.38±12.76 to 119.58±9.52) are comparable to those in other studies using the JSPE and conducted over the past 11 years [27]; however, they are higher compared to studies conducted in South Korea (105.90±12.8) [28], India (96.01±14.56) [29] and Iran (103.67±15.34) [30]. In addition, the present study found that the empathy scores up to year 3 were higher but slightly lower during the clinical years. Previous studies also suggest that empathy declines among medical students once they enter their clinical years or begin having contact with patients [31], [32].

According to a systematic review by Neumann et al. [10], several longitudinal studies and cross-sectional studies show a decline in empathy score as the student reaches more advanced levels of medical training. Hojat et al. [33] argued that this decline is due to increased cynicism and the erosion of idealism. Empathy, as measured by the JSPE, underlines the cognitive aspect [9]; consequently, the development of the affective and behavioural aspects of empathy, which are expected to gain more focus once students begin having contact with patients, may not be measurable through this tool. One study, which observed empathy among medical students in their clinical years, revealed that empathy actually increases during this time, despite a decrease in empathy scores as measured by a self-administered scale [34]. In addition, a review completed by Ponnamperuma et al. [35], suggests that studies on medical students’ empathy conducted in the Far East mostly show small but significant increases of empathy as the medical course progresses. 

Around 80-84% of the medical students at each year level were categorised into the HIHC motivation type (see table 1 (Tab. 1)). In another study, which involves medical students in a different setting, most of the students were categorised into the LIHC (31.8%) motivation type, followed by HILC (26.1%), HIHC (25.2%) and LILC (16.9%) [19]. According to the self-determination theory, the quantity of motivation does not always lead to the expected outcomes, especially when the quality is poor. High-quality motivation is determined by autonomous or internal control; conversely, externally controlled motivation is considered low quality [36]. 

The results of the present study reveal that most students were highly motivated to study medicine and showed great interest in the prestige of being a medical doctor [19]. Given the motivational profiles of both the preclinical and clinical students in the current setting, the present study suggests the importance of creating a nurturing environment in medical education, which should be able to fulfil students’ needs for autonomy, a feeling of effectiveness in their studies and the ability to relate to their peers [35].

The differences in empathy scores found in the present study may be explained by the variations in motivation type. Students with the HIHC type earned higher empathy scores compared to those of other motivation types, especially in first- and third-year preclinical year students and second-year clinical students (see table 2 (Tab. 2)). After calculating the correlation coefficients, a consistent association was found between motivation type and empathy score in these three year levels. The authors argue that medical students with the HIHC motivation type had higher scores in cognitive empathy, as measured by the JSPE, compared to those with other motivation types, as HIHC types apply both intellectual and affective empathy [13]. In collectivist and hierarchical cultures, as reflected in the current setting, HIHC types may be driven to achieve their intrinsic desires to become medical doctors while still attempting to fulfil external expectations by developing empathy towards patients, regardless of the patients’ conditions. 

Empathy is such a complex skill for medical students to learn, requiring a balanced development of cognitive, affective and behavioural components; nevertheless, it is considered a critical skill for future medical doctors and is thus highly valued by students and medical schools alike. According to the expectancy-value theory of motivation, the value of the goal towards which one is working and the expectation to achieve said goal are two out of four motivational factors moderating students’ self-efficacy [37]. The present authors suggest that the relationship between empathy (as a valuable task), self-efficacy and intrinsic motivation might explain the finding that empathy is positively correlated with the HIHC motivation type among medical students. 

This study may also lead to further discussion on the importance of a positive, nurturing and humanistic learning environment towards the development of empathy in medical students. Despite high levels of intrinsic motivation and self-efficacy, the complex task of understanding empathy and practising it in different clinical contexts necessitate a supportive learning environment, which encompasses personal development, goal progress, relationship and system maintenance, and change [38]. The learning environment is an inseparable part of the medical curriculum. In the present study, a consistent curricular approach combined with staff training focused on role-model development seemed to support the development of empathy and professionalism among medical students. 

### Limitations

This study was conducted at a single institution, which may limit its generalisability; however, the response rate was very good. In addition, because the analyses were completed at each year level throughout the preclinical and clinical stages, the specific characteristics of the students, curriculum and learning environment at each year level were considered when analysing the results. 

The authors are also aware that this study may be limited by its use of self-reported questionnaires; however, because the questionnaires were completed anonymously and did not carry any academic consequences for the students, the authors expect that the responses were honest. Further observations may be required to confirm the slightly lower empathy scores, as measured by the JSPE, during the clinical years. In addition, most students (80-84%) in this study fell into HIHC motivation type. With this result, the authors realised that other categories were not represented with an adequate number of students which may have had an adverse effect on the statistical analyses. 

Finally, the present study design did not allow the authors to explore causal relationships between students’ empathy and motivation type. In addition to the possible directions of relationships among the variables, it is important to understand the factors that influence empathy development among undergraduate medical students. 

## Conclusions

The present study provides empirical evidence of the associations between empathy and motivation type among medical students. Motivation type was found to be consistently associated with empathy score. The more the motivation profile is towards low intrinsic and low controlled, the lower the empathy score. Given that the medical students in the current setting mostly showed the HIHC motivation type, the findings also suggest the importance of exploring student motivations for entering medical school and evaluating them throughout their medical training. Further studies might explore the relationship between empathy and motivation type longitudinally and evaluate the role of learning environment in nurturing motivation and development of empathy among undergraduate medical students.

## Declarations

### Ethics approval and consent to participate

The study protocol was approved by the Research Ethics Committee of the Faculty of Medicine, Universitas Indonesia. All participants in this study approved of and provided their written consents. The participants were informed that their participation was completely voluntary and that the authors would ensure the confidentiality of the data provided in any of the reports that resulted from this study. 

#### Availability of data and material

The datasets generated and/or analysed during the current study are not publicly available but are available from the corresponding author on reasonable request.

#### Author contributions

Ardi Findyartini led the study, completed the analyses, developed the manuscript and confirmed the final version of the manuscript. Estivana Felaza collected the data, contributed to the development of the manuscript and confirmed the final version of the manuscript. Daniar Setyorini collected the data, completed the statistical analyses and contributed to the development of the manuscript. Rita Mustika completed the analyses, contributed to the development of the manuscript and confirmed the final version of the manuscript.

#### Authors’ information

- Ardi Findyartini is a senior lecturer of medical education in the Faculty of Medicine at Universitas Indonesia, where she heads the Department of Medical Education and serves as Chair of the Medical Education Center of IMERI.
- Estivana Felaza is a lecturer in the Department of Medical Education at Universitas Indonesia. She is also a researcher in the Medical Education Center of IMERI.
- Daniar Setyorini is a researcher in the Medical Education Center of IMERI at Universitas Indonesia.
- Rita Mustika a lecturer in the Department of Medical Education at Universitas Indonesia. She is also the Coordinator of collaborative projects at the Medical Education Center of IMERI.

## Funding

The study was funded by Universitas Indonesia through the scheme funding for international publication. 

## Acknowledgement

The authors would like to thank all the undergraduate medical students of FMUI who participated in the study, as well as James Wiguna Wahjudi, Andrew Kurniawan and Johan Qomarasandhy, who helped with data collection. The authors are also very grateful to Dr Retno Asti Werdhani, MEpid for her assistance in the statistical analyses, and colleagues in the Asia Pacific Medical Education Network (APME-net) for their constructive feedback on the study.

## Competing interests

The authors declare that they have no competing interests. 

## Figures and Tables

**Table 1:**
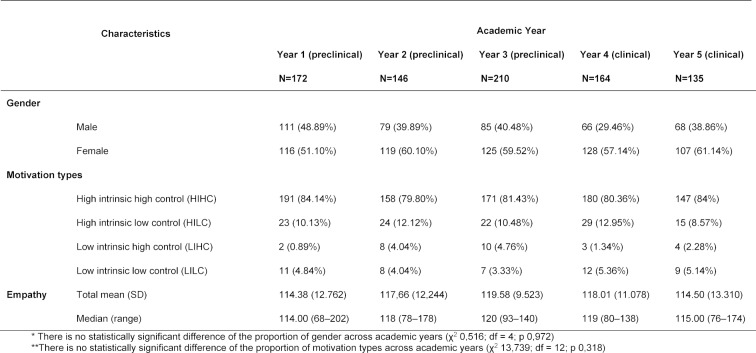
Characteristics of the respondents (n=827)

**Table 2:**
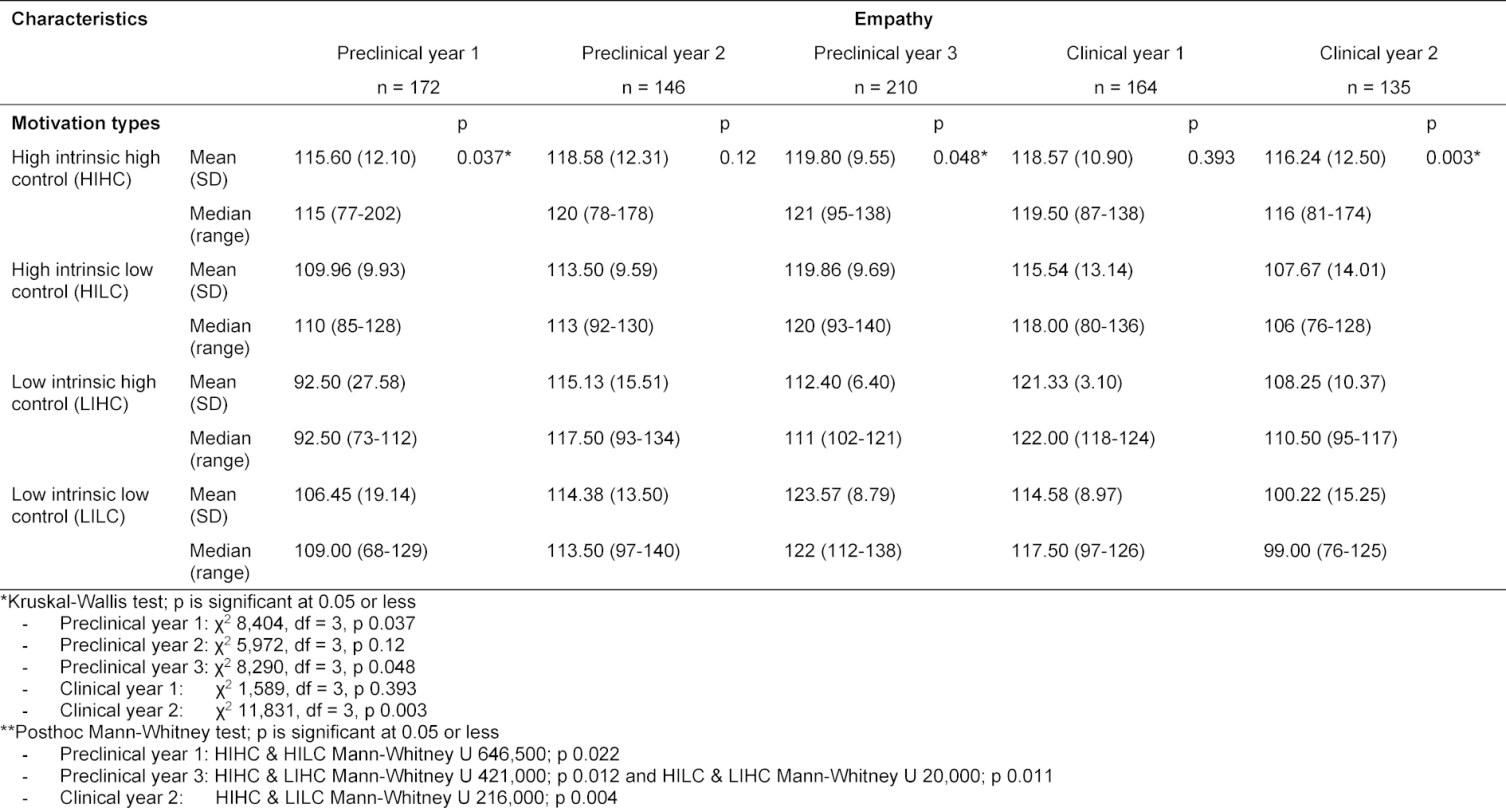
Empathy score according to motivation type (n=827)

**Table 3:**
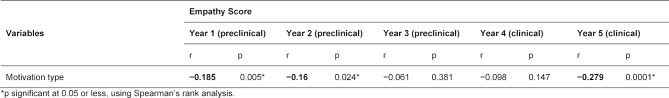
Associations between empathy and motivation (Spearman’s rank correlation coefficient)
